# Using the Blackburne-Peel index to measure the patellar height on magnetic resonance images

**DOI:** 10.1038/s41598-023-49497-0

**Published:** 2023-12-11

**Authors:** Volkan Kızılgöz

**Affiliations:** Department of Radiology, Faculty of Medicine, Erzincan Binali Yıldırım University, Erzincan, 24100 Turkey

**Keywords:** Magnetic resonance imaging, Orthopaedics

## Abstract

To reveal the normal values of patellar height measurements using the Blackburne-Peel index on magnetic resonance (MR) images and to show the gender and age differences regarding this measurement technique. This retrospective cross-sectional study investigated 148 knee MR images, and those images were re-evaluated to find out the normal values of patellar height using the Blackburne-Peel index (BPI). An adapted measurement technique of this index was applied to MR images. The study group was analyzed regarding the sex and age differences, correlation with age was investigated, and all descriptive data were presented. Independent Samples T-test, Kruskal–Wallis variance analysis, and Spearman’s Rho correlation test was applied to calculate the results. The mean value was 0.80 ± 0.09 for the total of patients. The measurements regarding the height of the inferior edge of the patella articular surface and the length of the patella articular surface indicated a significant difference between females and males (p < 0.001 for both measurements). The descriptive data obtained from this study revealed the mean values of the whole study group, different sexes, and age groups separately. Additional studies are needed for adapting BPI on MR images and verifying the normal values of the population.

## Introduction

Patellar height alterations may change the relationship between the patella articular surface and trochlea. This altered relationship leads to patellar instability, a common but complex knee condition with the highest incidence reported in 14- to 18-year-old patients^1^. Patellar height is one of the parameters measured for patellar instability to diagnose patella alta or baja, which plays a role in this condition. Patellar height measurements and classifying the trochlear morphology help guide clinical and surgical decision-making^2^.

Blackburne-Peel index (BPI) is a commonly used method for assessing patellar height^3^. BPI is an alternative to the Insall–Salvati index to diagnose patella alta and baja. Some authors in the literature indicate that BPI has higher interobserver reliability compared to the Insall–Salvati index. Moreover, BPI can also be used when an abnormality is observed in tibial tuberosity (e.g., osteotomies or Osgood-Schlatter disease) and has the advantage of not relying on this trademark, unlike the measurement method of Insall–Salvati index^4,5^.

Magnetic resonance (MR) imaging is widely used to diagnose various conditions and renders the assessment of multiple parameters in the knee. One may simultaneously evaluate multiple parameters at the same image and provide quick data for different conditions. The most common methods used in assessing patellar height are based on the measurements on the lateral knee radiographs. It would be beneficial to use these indexes on MR images for a quick evaluation of patellar height and to consider this data with other lesions or signal alterations.

There are papers applying the measurement techniques of these indexes on MR images^6^; however, the measurement values on MR images may reflect different results compared to radiographs^3^. In this current study, the adaptation of this method to MR images was discussed, and the normal values of the BPI index with gender and age group comparisons were presented.

## Materials and methods

The institutional ethics committee (Erzincan Binali Yıldırım University, Ethics Committee of Clinical Research) approved this retrospective cross-sectional study. Due to the retrospective nature of the research, the ethics committee (Erzincan Binali Yıldırım University, Ethics Committee of Clinical Research) has waived the need for informed consent from each patient enrolled in the study. After the ethics committee approval, MR images of all patients who underwent knee MR imaging between 1 and 31 of January 2023 were re-evaluated regarding the inclusion and exclusion criteria of the investigation. All methods used in this research were performed in accordance with the relevant guidelines and regulations.

### Patients

**Figure 1:**
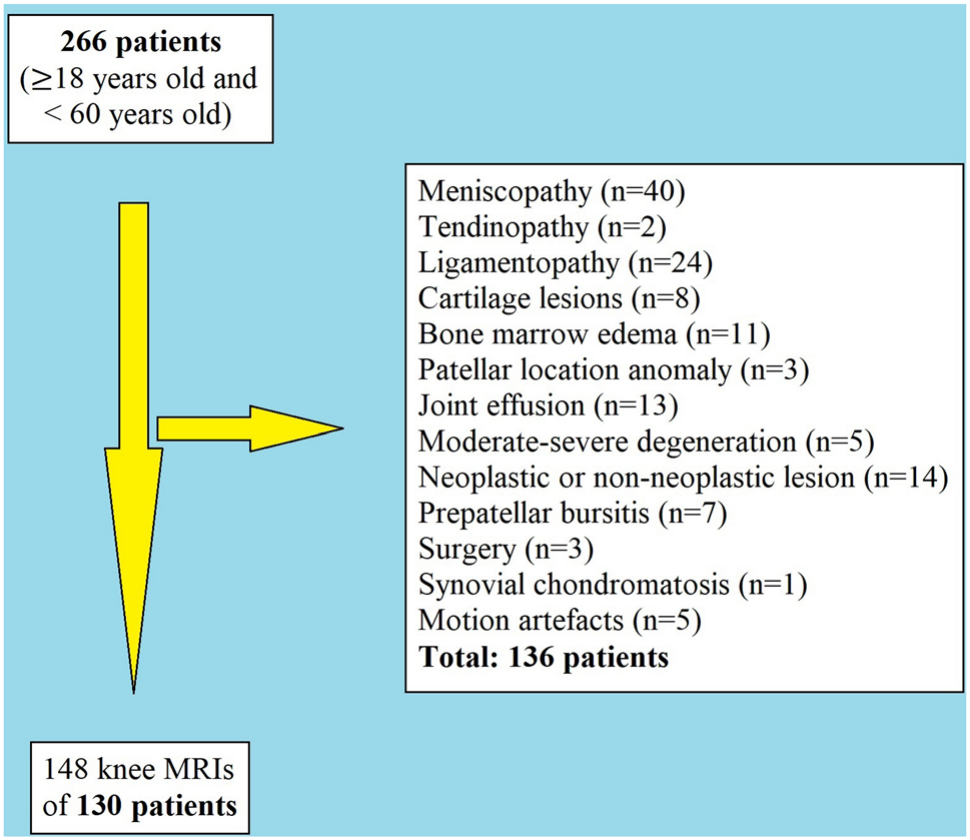
The workflow of the study. Some excluded patients had more than one exclusion criteria, and in this flowchart, these patients were classified with the most important pathology relevant to clinical symptoms.

To avoid the effect of degenerative arthritis on the results of the study and to find the normal values of patellar location in skeletally mature individuals, patients aged between 18 and 60 years old were enrolled in this investigation. These age limits were entered in the picture archiving and communications system (PACS), and 266 patients were listed in this specific period. MR images with any lesions that may play a role in kinematic alteration of the knee were tried to be avoided to find the normal values of patellar location with BPI measurements. The patients with any type of meniscus tears (n = 40), patellar tendinopathies (n = 2), any ligamentous signal change or ruptures including anterior, posterior cruciate ligaments or collateral ligaments of the knee (n = 24), any cartilage defects or chondromalacias (n = 8), any knee MR images with bone marrow edema which be suspicious for micro trabecular fractures (n = 11), patellar location anomalies (2 patients with patella baja and one patient with patella alta), patients with joint effusions which may be a result of infectious or a rheumatologic processes (n = 13), five patients with moderate and severe degenerative arthritis (Kellgren–Lawrence type 3 and 4), neoplastic or non-neoplastic lesions (including osteochondritis dissecans, intraarticular synovial cysts, enchondromas, etc.) around the patellofemoral joint or articular plateau of the tibia (n = 14), seven patients with prepatellar bursitis, any kind of knee surgeries (n = 3), and one patient with synovial chondromatosis were excluded from the study. Vertical malalignment and other patellar anomalies, such as bipartite patella or transient patellar dislocation, were also planned to be excluded; however, there was no patient in this situation among the patients enrolled in the study. Five patients with knee MR images that cannot be appropriately interpreted due to motion artifacts were also excluded. Totally 130 of 266 patients were re-assessed regarding patellar positions using BPI measurements. 18 patients (13 women and 5 men) had bilateral knee MR examinations in the study population (Fig. 1).

### Magnetic resonance imaging

A 1.5 T MR machine (Magnetom Aera, Siemens, Erlangen, Germany) with standard (15-channel) knee coils was used to perform knee MR imaging. Each patient was examined in supine position with slight flexion (ranging between 5° and 15°) of the knee and approximately 15° of external rotation. The imaging process started above the trochlear groove, all of the suprapatellar joint recess was included in the field of view and the imaging planes were extended to the area below the tibial tubercle. To obtain the knee MR images, turbo spin echo technique with different sequences was applied. Sagittal plane T1 weighted imaging (time of repetition [TR], 1110 ms; time of echo [TE], 9.7 ms; field of view [FOV], 180 mm; slice thickness [ST], 3 mm; number of excitations [NEX], 1; voxel size [VS], 0.5 × 0.5 × 3 mm), fat-saturated proton density imaging in coronal plane (TR, 2390 ms; TE, 20 ms; FOV, 200 mm; ST, 4 mm; NEX, 2; VS, 0.6 × 0.6 × 4 mm), proton density-weighted fat-saturated imaging in axial plane (TR, 3440 ms; TE, 30 ms; FOV, 180 mm; ST, 3 mm; NEX, 2; VS, 0.6 × 0.6 × 3 mm), fat-saturated proton density imaging in the sagittal plane (TR, 3060 ms; TE, 38 ms; FOV, 180 mm; ST, 3 mm; NEX, 1; VS, 0.7 × 0.7 × 3 mm) were used for each patient.

### Interpretation and measurements

**Figure 2:**
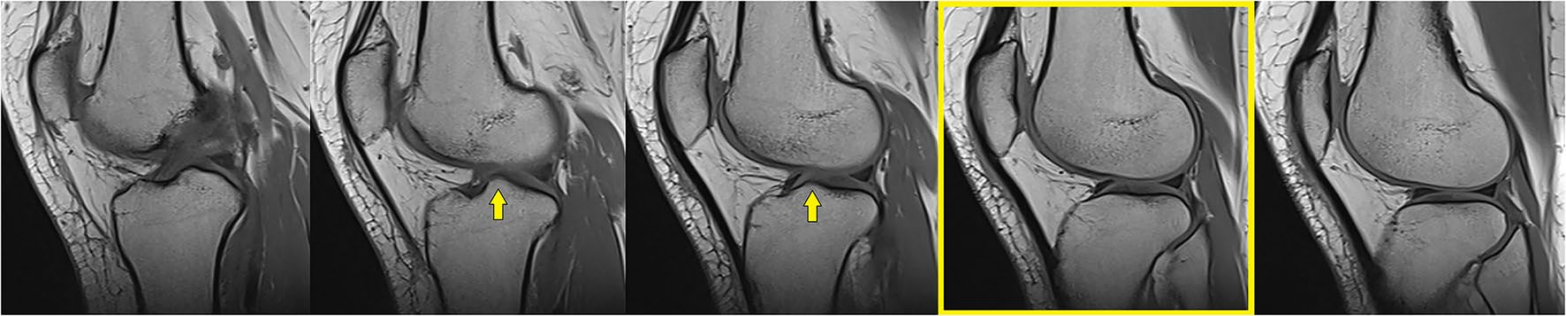
Five consecutive mid-sagittal planes in T1 weighted sequence. The adjacent sagittal plane to the lateral tibial tubercle in which the tubercle (yellow arrows) cannot be visualized was chosen to measure the patellar height (shown in the yellow frame).

**Figure 3:**
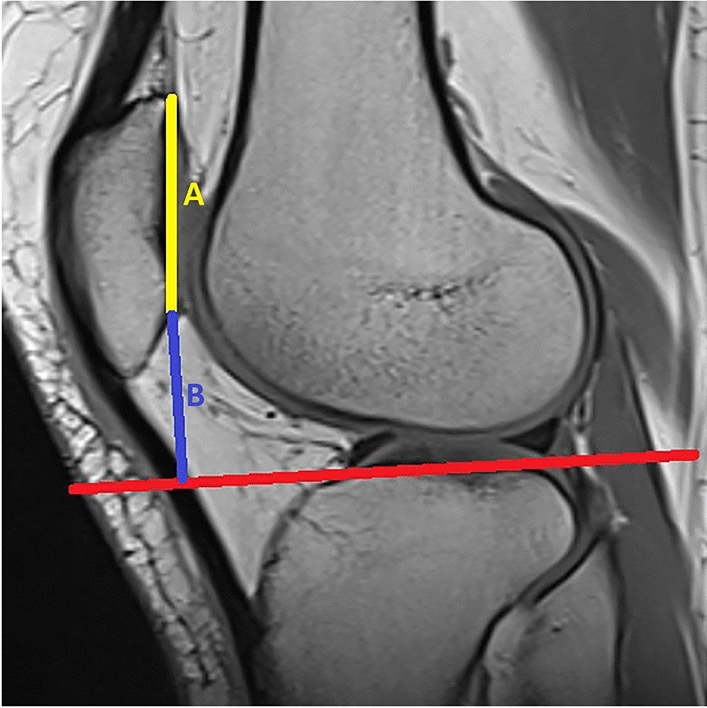
The line that passes through the flat surface of the lateral tibia articular plateau was accepted to be the baseline (red line). The ratio of the distance between the most inferior edge of the patellar articular surface and this baseline (**B**) to the line passes along the surface of the patella articular surface (**A**) was calculated as the Blackburne-Peel index.

All MR images were reviewed by a musculoskeletal radiologist with 17 years of experience. A PACS system (Akgün PACS Viewer v7.5, Akgün Software, Ankara, Turkey) was used to analyze the cross-sectional images in standard digital imaging and communications in medicine (DICOM) formats. T1 weighted sagittal planes were used to measure the patellar height regarding the Blackburne-Peel index. To use this method on MR images properly, the sagittal image in which the patellar articular surface and flat surface of the tibial plateau were needed to be on the same image. The mid-sagittal planes were controlled for each patient, and the adjacent sagittal plane to the lateral tibial tubercle (eminence), which the tubercle cannot be visualized, was chosen to measure the patellar height (Fig. 2). In this image, the line passing through the flat surface of the lateral tibia articular plateau was accepted as the baseline. To determine this baseline, all patients were measured using the flattened cortex of the lateral tibial plateau in this sagittal image. The baseline was extended anteriorly to measure the distance between the inferior patellar edge and the baseline. The ratio of the distance between the most inferior edge of the patellar articular surface and this baseline to the length of the articular surface of the patella was calculated as BPI (Fig. 3). All the patients were measured twice, and the average value of these measurements was accepted as the final result. Every measurement was noted in millimeters, using two digits after the comma.

### Statistical analysis

All statistical calculations were applied using IBM SPSS Statistics for Windows version 22.0 (IBM Corp., Armonk, NY, USA). The data distribution characteristics were checked by Shapiro–Wilk tests. Independent samples T-tests were carried out to compare females and males regarding the length of the patellar articular surface (A), the height of the most inferior edge of the patella articular surface from baseline (B), and BPI values. Comparison of age groups regarding B, A, and BPI values was carried out by Kruskal Wallis variance analysis. Spearman’s Rho test was used to investigate any correlation with B, A, and BPI values. The p-values < 0.05 were considered to indicate the significance of the difference in all statistical calculations.

### Ethics

This study was approved by the institutional ethics committee (Erzincan Binali Yıldırım Universitesi, Tıp Fakültesi Dekanlığı, Klinik Araştırmalar Etik Kurulu, Erzincan/Turkey, Number: 2023-14/13, Session: 14, Date: 10.08.2023).

## Results

The height of the most inferior edge of the patella articular surface from baseline, length of the patellar articular surface, and BPI values showed normal data distribution; thus, parametric tests could be applied. However, the number of patients in the 50–59 age group (n = 10) was insufficient to perform a parametric test for statistical calculations regarding age group comparisons.

There were 130 patients (84 female, and 46 male, mean age 33.91 ± 10.28) enrolled in this study. The demographic data of the study population is presented in Table 1. There was a significant difference between sex groups regarding the distance between the most inferior edge of the patellar articular surface baseline of the tibia articular surface (B) and the length of the patella articular surface (A) (*p* < 0.001 for both values). However, no significant difference was observed between female and male groups regarding BPI.

**Table 1:** Demographic properties of the study population.

		n		%	
Sex					
Females		84		64.6	
Males		46		35.4	
Age groups (years)					
18–29		52		40.0	
30–39		37		28.5	
40–49		31		23.8	
50–59		10		7.7	

		Mean		Standard deviation	
Mean values of age and measurements					
Age (years)		33.91		10.28	
B		24.84 mm		3.02	
A		31.06 mm		2.36	
BPI		0.80		0.09	

	Age groups (years)				Total
	18–29	30–39	40–49	50–59	
Crosstabulation of sex and age					
Sex					
Females					
n	29	21	24	10	84
% within sex	34.5%	25.0%	28.6%	11.9%	100.0%
% within age group	55.8%	56.8%	77.4%	100.0%	64.6%
Males					
n	23	16	7	0	46
% within sex	50.0%	34.8%	15.2%	0.0%	100.0%
% within age group	44.2%	43.2%	22.6%	0.0%	35.4%
Total					
n	52	37	31	10	130
% within sex	40.0%	28.5%	23.8%	7.7%	100.0%
% within age group	100.0%	100.0%	100.0%	100.0%	100.0%

B: The distance between the most inferior edge of the patellar articular surface baseline of the tibia articular surface, A: the length of the patella articular surface.

*BPI* Blackburne-Peel index.

**Figure 4:**
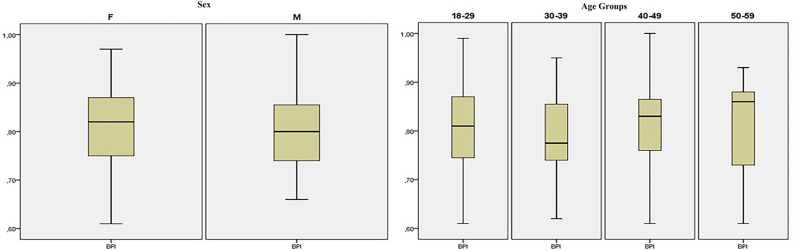
The data distributions of Blackburne-Peel index measurements on magnetic resonance images regarding sex and age groups.

A significant difference was indicated between the different age groups in the study regarding the A values (*p* = 0.003). On the other hand, there was no difference between these groups regarding BPI measurements (*p* = 0.333) (Table 2). Although no difference was indicated between the age groups, a Dunn Test revealed significantly lower values for patients in the 50–59 age group compared to 18–29 and 30–39 age groups regarding the BPI values. The B value showed a significant, negative, and very low-level correlation (*p* = 0.045), while the A value indicated a significant, negative, and low-level correlation (*p* = 0.002). However, no correlation was found between age and BPI values (*p* = 0.767). The data distributions regarding sex and age groups are presented in Fig. 4.

**Table 2:** Comparisons between sex and age groups regarding B, A, and BPI measurements on MR imaging.

Sex	n	Mean ± SD (mm)	*p* value
Comparisons regarding sex groups			
B value			
Females	97	24.17 ± 2.69	** < 0.001**
Males	51	26.40 ± 2.89	
A value			
Females	97	30.04 ± 1.79	** < 0.001**
Males	51	32.92 ± 2.29	
BPI			
Females	97	0.80 ± 0.08	0.924
Males	51	0.80 ± 0.09	
Comparisons regarding age groups			
B value			
18–29	60	25.49 ± 3.10	0.153
30–39	40	24.64 ± 2.95	
40–49	35	24.79 ± 2.89	
50–59	13	23.69 ± 1.99	
A value			
18–29	60	31.48 ± 2.15^α^	**0.003**
30–39	40	31.54 ± 2.89^α^	
40–49	35	30.36 ± 1.83	
50–59	13	29.25 ± 2.17^β^	
BPI			
18–29	60	0.81 ± 0.09	0.333
30–39	40	0.78 ± 0.08	
40–49	35	0.81 ± 0.09	
50–59	13	0.81 ± 0.09	

B value: The distance between the most inferior edge of the patellar articular surface baseline of the tibia articular surface, A value: the length of the patella articular surface.

*BPI* Blackburne-Peel index, *SD* standard deviation.

^α^Statistically higher values compared to ^β^according to pairwise comparisons.

Significant values are in bold.

## Discussion

This research indicated the mean BPI values of different age groups, females, males, and the whole study population using MR images. The age and gender differences were studied, and no significant difference was observed regarding these parameters. Additionally, no correlation was found between age and BPI measurements.

The commonly used indexes to measure the patellar height were obtained by lateral radiographs since the bony anatomy of the patella and the adjacent structures, which forms the anterior compartment of the knee, were defined easily with this imaging modality. However, MR imaging is widely used to depict various conditions of the knee, presenting the cartilaginous, ligamentous, and bony anatomy together in a frame. MR imaging provides a detailed assessment to indicate the results of multi-factorial effects on the knee. Adapting the measurement methods of the patellar height on MR images would render a broader evaluation spectrum of the patella-femoral joint diseases and provide a quick assessment of routine knee MR image series.

The Insall–Salvati index is one of the commonly used measurement methods to reveal the patellar height and provides data by measuring the patellar tendon and the superior–inferior length of the patella. Some problematic situations prevent accurate measurement of these structures where the tibial tuberosity (e.g., Osgood–Schlatter disease or osteotomies) or the inferior pole of the patella (Sinding–Larsen–Johansson) is affected. On the other hand, the Blackburne-Peel index uses the tibia articular plateau and patellar articular surface, and this method is not affected by such conditions mentioned above. Some authors claim that BPI was more accurate and reproducible than the Insall–Salvati index to indicate the patellar height^7^.

Some authors declared lower inter observer reliability on MR images, compared with knee X-ray results regarding the BPI measurements^3,8^. This situation is thought to be a possible result of observing non-flat tibia articular plateau on sagittal MR images which creates a difficulty when drawing a straight baseline^3,9^. To measure the BPI, drawing the baseline passes through the tibia articular plateau is crucial to determine the index value. The awareness of specific properties of the tibia articular plateau is essential to understand this measurement technique better. Considering that the surface of the lateral tibial articular plateau is more flat than the relatively concave-shaped surface of the medial articular plateau^10^, the lateral side of the articular plateau is more likely to contribute to the baseline drawn in the lateral knee images. Besides, the lateral side of the patella is wider than the medial side, which is meaningful considering the anatomic alignment of the bony elements of the patella-femoral joint^11^. In addition, the tibial tubercle would be expected to change the BPI value by altering the natural baseline of the flat tibia articular plateau and providing an increased slope to the baseline. Thus, the adjacent sagittal slice to the lateral tibial tubercle in which the tubercle cannot be visualized was chosen to measure the patellar height on MR images to reflect a better infrastructure for BPI measurements in this study.

In the study of Blackburne and Peel, the mean index values of females (0.806 ± 0.13) and males (0.805 ± 0.14) with normal knees were lower than the females (1.01 ± 0.25) and males (0.92 ± 0.21) with recurrent patellar subluxation^12^. Studies determining BPI using lateral radiographs among normal adult populations obtained different results in different regions. In a study conducted among the Indonesian population (n = 136), the mean values of BPI measurements have been revealed as 0.90 ± 0.10, 0.90 ± 0.09, and 0.89 ± 0.10 for the total study population, females, and males, respectively^13^. In another study for normal individuals of an Indian population (n = 100), BPI values were 0.67 ± 0.02 and 0.67 ± 0.01 for females and males, respectively^7^. There is not much in the literature mentioning the average BPI values obtained by MR imaging. However, some studies measured BPI for different reasons and compared the patients with certain knee conditions to the control groups. Loose et al. studied the alterations for patellar height in patients with and without patellar tendinopathies (PT). The mean BPI values of patients with PT (n = 38) was 1.14 ± 0.18, and patients without PT were 1.07 ± 0.16 on MR images^6^. In Yue et al.’s research, the BPI values of the study group (n = 112) consisting of patients with lateral patellar dislocation (LPD) were compared control group consisting of patients without patellofemoral instability (n = 129). The mean values obtained by MR images for females and males were 1.10 ± 0.19 and 1.05 ± 0.19, respectively, in the control group, and no significant difference was observed between genders. However, in the same study, the BPI values obtained by lateral knee radiographs were 0.89 ± 0.18 and 0.94 ± 0.18 for females and males, respectively. As the authors indicated in this investigation, MR imaging demonstrated higher values than knee radiographs regarding BPI measurements^3^. The current study results indicated the mean values for females and males as 0.80 ± 0.08 and 0.80 ± 0.09, respectively.

The difference in the results of BPI measurements between lateral knee radiographs and MR imaging might result from a weight-bearing versus a non-weight-bearing imaging modality. As discussed in Yue et al.’s study, the quadriceps contraction would cause the patella to move proximally, and BPI would be measured higher in weight-bearing imaging. However, the results of their investigation revealed the opposite; the BPI values were higher than radiographs and made the authors propose that weight bearing does not have a significant effect on this situation^3^.

Since not much study mentions the average values of BPI on MR imaging, the sex differences regarding this measurement method are difficult to compare with the results of the current study. Yue et al.’s control group measured in their study (n = 129) revealed no significant difference between females and males (p = 0.198) regarding BPI values. The current study results indicated no difference between sex groups regarding BPI values, similar to this study. Besides the gender difference, age was another parameter to be discussed in a possible relationship with patellar height. The loss of strength and atrophy in the patellar tendon and quadriceps muscle fibers due to aging, which cannot be visualized on MR imaging, might play a role in altered patellar height. Therefore, the patients enrolled in the study were grouped regarding age, and the results were also analyzed regarding this aspect. There is not much in the literature presenting normal values of the age groups. In Sanjeev Joshi et al.’s research, age groups between 20 and 64 years were classified into 5-year sections. The significance of the difference was not underlined in this study, but relatively higher BPI values were presented in this investigation regarding the measurements on lateral knee radiographs. When the age groups were considered, the current study revealed no significant difference regarding BPI values obtained on MR images. On the other hand, a pairwise comparison test indicated significantly lower values for patients in the 50–59 age group compared to 18–29 and 30–39 age groups regarding BPI measurements. In addition, age showed no correlation with BPI values in this current study.

A careful evaluation of the results in this current study is necessary since there are several limitations to discuss. First, this research dataset belongs to a single center and a specific population which may differ from other regions of the world. The genetic factor is an important aspect to consider the normal BPI values. Even though the measurements are performed by a musculoskeletal radiologist with 18 years of experience, two or more reviewers would be much better to provide more accurate data and present the BPI’s inter-observer reliabilities. Third, BPI measurements were identified on lateral knee radiographs with a 30° of knee flexion, and this angulation is underestimated in this study to use this measurement technique on the routine knee MR images. In addition to all these facts, some authors in the literature indicate the effect of the tibial slope on BPI measurements, claiming that BPI values decrease with the increasing slope^14^. Lateral slope measurements were not performed in this current study and this relationship was another aspect that needs to be considered, which might influence the study results of this article. More investigations comparing the BPI results of patients with relatively high and low lateral slope values might be useful to understand this relationship better.

## Conclusion

The Blackburne-Peel index would be beneficial to use on MR images, especially to determine patellar height quickly with associated anomalies or lesions on routine knee MR imaging without the necessity of any other imaging modalities. Interpretation of the measurement results depends on the knowledge and awareness of the standard values of the population. Enlarged study populations from different geographical regions are expected to contribute positively to this adapted measurement technique of BPI used in this current research on MR images.

## Data Availability

The datasets generated and/or analyzed during the current study are not publicly available due to the risk of a breach of patient data privacy. However, anonymized data are available from the corresponding author upon reasonable request.
